# Everything everywhere all at once: mapping lay beliefs about self-control

**DOI:** 10.3389/fpsyg.2025.1593089

**Published:** 2025-05-01

**Authors:** Jinyao Li, Marleen Gillebaart, Tim van Timmeren, Denise de Ridder

**Affiliations:** Department of Social, Health, and Organisational Psychology, Utrecht University, Utrecht, Netherlands

**Keywords:** self-control, willpower, lay beliefs, trait self-control, open questions

## Abstract

**Introduction:**

Self-control is essential for achieving long-term goals and is influenced by individuals’ beliefs about it. Previous studies have found that those who view self-control as non-limited perform better in cognitive tasks, resist temptations more effectively, and achieve better outcomes. Understanding these beliefs is therefore crucial for fostering stronger self-control, yet a systematic understanding remains lacking.

**Methods:**

To comprehensively map these beliefs, participants from the United States, the Netherlands, and China (total *N* = 150) were directly asked about their views on self-control.

**Results:**

By analyzing these responses, we identified 14 key components of self-control beliefs. These beliefs highlight the challenging, committed, and disciplined nature of self-control, offering a broader perspective beyond the traditional view of self-control as a limited resource.

**Discussion:**

Findings provide a comprehensive framework for understanding lay beliefs about self-control and underscore their significance in shaping self-control exertion.

## 1 Introduction

Humans need self-control almost every day, from resisting an appealing but unhealthy meal to curbing their urge to watch Netflix episode after episode at midnight. Self-control is generally defined as the ability to override one’s inner responses, as well as to interrupt undesired behavioral tendencies (such as impulses) and refrain from acting on them (Hofmann et al., 2014; Tangney et al., 2004). Self-control extends its influence across various domains of life, including school and work performance, health, personal finance, and interpersonal relations, thus affecting people’s well-being and success in life (De Ridder et al., 2012; Tangney et al., 2004).

Numerous scholars have focused on uncovering the underpinnings of self-control in the past decades, leading to a rich yet multifaceted array of perspectives on how self-control operates. These perspectives have engendered discussions centered on dimensions such as inhibition versus initiation (Cheung et al., 2014; De Ridder and Gillebaart, 2017; Hofmann et al., 2012), effortful versus effortless exertion (Galla and Duckworth, 2015; Gillebaart and de Ridder, 2015), strategic and “smart” self-control (Duckworth et al., 2016; Fujita et al., 2020; Milyavskaya et al., 2021), and flexibility (Bürgler et al., 2021; Werner and Ford, 2023). As a result, a contemporary perspective of self-control has emerged that allows for effortful inhibition, initiation, effortless strategies, flexibility, and automatic behavior to all play a role in self-control behavior.

While these viewpoints offer valuable insights into distinct aspects of self-control, they consistently consider self-control as a capacity, focusing on (boundary) conditions under which self-control is successful. This overlooks a relevant and understudied aspect of self-control, that is, how self-control is perceived by people, as determined by their lay beliefs about self-control. To fill this gap, we posit a belief-approach to the problem of divergent theoretical stances in self-control research. This approach may contribute to our understanding of different perspectives on self-control, as how self-control is experienced, utilized, and exerted may hinge on an individual’s beliefs about it. The multiple self-control perspectives may then coexist rather than compete, considering that they may reflect different views of self-control held by people, which, in turn, may influence their self-control exertion. Therefore, the current study seeks to map lay beliefs on self-control as a necessary initial step to foster our understanding of the role of humans’ beliefs about self-control.

Lay beliefs, understood as what people believe about the characteristics of things, places, and other people (Dweck and Leggett, 1988; Ross and Nisbett, 2011), have shown to have considerable effects on guiding people’s cognition, affect, motivation, and action (Molden and Dweck, 2006). For instance, people may hold beliefs about whether intelligence (Dweck and Leggett, 1988) and emotion (Tamir et al., 2007) is fixed or malleable. Such beliefs can be impactful for their goal performance, well-being and social adjustment (Dweck and Leggett, 1988; Tamir et al., 2007). People may also hold beliefs about a just world, which motivates them to strive for long-term goals (Lerner, 1980). In our research, we understand self-control beliefs as a broad set of ideas and thoughts people may have about self-control. They can be about the nature of self-control and how self-control operates.

Previous research has already identified several beliefs related to self-control and has shown how they may impact individual’s self-control performance. Job et al. (2010, 2015), for example, found that the belief whether self-control is either or not a limited resource, affects self-control performance on demanding tasks, such as exam performance: those endorsing limited self-control beliefs exhibited poorer performance compared to those embracing an unlimited perspective. Similarly, individuals who believe self-control is malleable and unlimited tend to set more goals, compared to those who think self-control is fixed and limited (Mukhopadhyay and Johar, 2005).

Furthermore, people may hold beliefs regarding emotions and strategies associated with self-control, which can affect their self-control decisions. Evidence suggests that individuals with high trait self-control particularly are likely to believe that positive emotions, such as pride and hope, are more useful for self-control success than negative emotions (Tornquist and Miles, 2019). Recent research comparing beliefs about willpower—the capacity to resist temptations and thus often equated with self-control—and self-control strategies suggests that people generally perceive individuals with high trait self-control as having greater willpower rather than better self-control strategies (Gennara et al., 2023). Moreover, situational strategies are considered more peripheral in people’s understanding of the nature of self-control (Bermúdez et al., 2023).

These studies suggest that people hold multiple beliefs about the nature of self-control, encompassing diverse aspects such as its availability, malleability, related emotions, strategies, and morality (Mooijman et al., 2018). Moreover, these beliefs can significantly affect people’s self-control performance. However, these beliefs have largely been studied in isolation, with research either examining a single belief (e.g., Francis et al., 2023; Jankowski and Job, 2023; Job et al., 2010) at a time or comparing two beliefs (e.g., Gennara et al., 2023; Mukhopadhyay and Yeung, 2010) to align with existing scholarly notions of self-control. In addition, beliefs were often manipulated rather than assessed (e.g., Garrison et al., 2023; Gennara et al., 2023), leaving uncertainty about their prevalence and endorsement among the general public. Hence, we posit that a systematic investigation of self-control beliefs is necessary, particularly from a more ecologically valid bottom-up approach in which people are asked directly about their self-control beliefs.

Given that beliefs might be shaped by the cultural context in which they emerge, we will map self-control beliefs across cultures. Previous research suggests that a cross-cultural perspective gives a better picture of diverse beliefs held by individuals from various cultural backgrounds. For instance, a comparative study involving Japanese and American children found variations in delayed gratification behaviors, which may be attributed to variances in their beliefs (Yanaoka et al., 2022). Similar to self-control, delayed gratification emphasizes inhibitory control and the ability to restrain impulses. Yanaoka et al. (2022) found Japanese children displayed better self-control in delaying gratification for food, while American children exhibited stronger self-control by delaying gratification for opening gifts. These disparities may stem from distinct cultural habits and norms, suggesting that self-control is driven by ideas about when delay of gratification is appropriate, whether for food or for opening gifts (Yanaoka et al., 2022). Similarly, a study comparing participants from India, the United States, and Switzerland found that self-control was viewed differently across cultures (Savani and Job, 2017). Americans and the Swiss viewed self-control exertion as an exhausting experience, whereas in India, where cultural values emphasize the virtue of self-control exertion, it was seen as an energizing experience. Importantly, these differences in beliefs about self-control affected people’s self-control exertion (Savani and Job, 2017). Therefore, examining beliefs in their cultural context may thus give more opportunities to comprehensively reveal the ideas people hold about self-control.

Based on the above, the primary aim of the current study is to map diverse self-control beliefs people hold. As a secondary goal, we looked at how these beliefs might vary contextually and individually by associating them with cultural backgrounds and individual backgrounds (e.g., age and gender). We also examined associations of self-control beliefs with trait self-control so as to relate the capacity for self-control with understandings of self-control.

Building on potential variances in self-control beliefs among American, Asian, and European cultures (Savani and Job, 2017; Sun et al., 2019; Yanaoka et al., 2022), we recruited participants from three countries representing three continents: the United States, China, and the Netherlands. People in these three countries have been shown to vary in cultural dimensions related to self-control, such as motivation towards achievement and success, long-term orientation, and indulgence (Hofstede, 2011). We expected that those variances in general cultural dimensions may be reflected in individuals’ beliefs regarding self-control.

To achieve a comprehensive understanding of self-control beliefs, we took a bottom-up approach. Specifically, we used open-ended questions to directly examine participants’ beliefs on self-control. To identify a broad range of beliefs people have about self-control, we held no a priori expectations regarding the number and nature of beliefs people would report. This study was approved by the Ethics Review Board of the Faculty of Social and Behavioural Sciences, Utrecht University, and filed under number 23–0151. All data and materials are available at OSF.^1^

## 2 Method

### 2.1 Participants and procedure

A total of 150 participants from the US, the Netherlands, and China (i.e., 50 from each country), were recruited online on Prolific (for US and the Netherlands) and Credamo (for China). This sample size was deemed adequate to reach data saturation using an open-ended questionnaire (Tran et al., 2016), for gathering a wide range of self-control beliefs.

The total sample’s mean age was 31.16 (*SD* = 9.07) years, with the US, the Netherlands, and China samples reporting mean ages of 34.60, 28.70, and 30.18 years, respectively. Of the whole sample, 45.3% were male (52, 50, and 34% in US, Netherlands, and China, respectively) and 77.3% had graduated from college (54% in US, 78% in the Netherlands, and 100% in China). See Table 1 for demographical details.

**Table 1:** Demographical characteristics of participants (*N* = 150).

Demographic Characteristics	US	NL	CN	Full sample
Age	34.60	28.70	30.18	31.16 (9.07)
Gender				
Female	22	25	33	80
Male	26	25	17	68
Others	2	0	0	2
Highest education				
High school	23 (46%)	11 (22%)	0 (0%)	34 (23%)
College	27 (54%)	39 (78%)	50 (100%)	116 (77%)

*N* = 150, and 50 for each of the countries, the United States (US), the Netherlands (NL), and China (CN); participants’ highest education levels (*p* = 0.005) and ages (*p* = 0.003) differed significantly across the three countries.

Prior to taking part in the study, participants received information about the study and provided informed consent. Next, they proceeded with measures assessing their self-control beliefs and trait self-control and filled out demographics in their native languages (i.e., English, Dutch, or Chinese).^2^ No time limit was imposed on participants. Participants were reimbursed with (an equivalent of) $1.26 (for US and the Netherlands) for finishing the study. Chinese participants received a similar amount of money ($0.28) in terms of spending capacity and platform’s guidelines.

### 2.2 Measures

#### 2.2.1 Self-control beliefs

An open-ended questionnaire was used to assess individuals’ self-control beliefs. Specifically, participants were asked to provide five words to complete the sentence “*In my view, self-control is…*” (*cf.* Clifton et al., 2019). The term *Self-Control* was introduced to participants at the beginning of the questionnaire, accompanied by examples (e.g., a short-term temptation of eating a chocolate bar versus a long-term goal of a healthy body weight). Following this, and prior to being asked about their thoughts on self-control, participants were presented with specific examples of self-control dilemmas and then asked to recall and write down their own experiences with such dilemmas to prompt their ideas about self-control.

The questionnaire was initially created in English and translated into Dutch and Chinese following cross-cultural translation norms (Brislin, 1970). Bilingual experts with fluency in English and/or Dutch and/or Chinese carried out the translation and back-translation procedures.

#### 2.2.2 Trait self-control (TSC)

The 13-item Brief Self-Control Scale (BSCS) by Tangney et al. (2004) was used to measure trait self-control. The Chinese version of BSCS was from Unger et al. (2016). The scale employs a 5-point Likert scale where 1 represents *not at all* and 5 represents *very much*. A higher score reflects a higher level of trait self-control. An item example is “*People would say that I have iron self-discipline.*” The scale proved reliable in the study, with Cronbach’s alphas of 0.89, 0.84, and 0.94 for American, Dutch, and Chinese participants, respectively.

Finally, demographical information including gender, age, and education was assessed with single questions.

## 3 Analysis

We analyzed participants’ responses to the question about self-control beliefs by means of open coding (Williams and Moser, 2019), without any a priori assumptions about the number or nature of self-control components. To prepare for the analyses, Dutch and Chinese responses were first translated into English by ChatGPT 3.5 for further comparison and integration.^3^ Moreover, responses from the three countries were randomized to blind country information for further analyses. We then conducted our analyses in the following consecutive steps. All data were coded by four authors; details of the coding process are summarized in Table 2.

**Table 2:** Processes of generating components of self-control beliefs.

Steps	Method of analysis
Step 1: Data preparation and familiarization	We translated the responses into English using ChatGPT 3.5, randomized the order to blind country information, and familiarized ourselves with the data by reading and re-reading the responses. We took notes and verified the accuracy of translations.
Step 2: Word list generation	We used NVivo 14 to generate a comprehensive list of words appearing in the responses.
Step 3: Word themes formation	We condensed the word list into word themes by merging different word forms (e.g., “difficulty” and “difficulties”) and synonyms (e.g., “difficulty” and “hardness”). One author conducted this process, and all authors reviewed and discussed it.
Step 4: Initial components development	We collaboratively identified an initial set of self-control belief components based on the word themes. These components were developed through rounds of discussion, capturing underlying conceptual similarities and the core meaning of the word themes. One author independently verified the components.
Step 5: Coding all responses and refining components	This coding step was highly iterative and involved two rounds: (1) We coded all 750 responses into the generated self-control belief components, leaving any responses that caused hesitation for the next round; (2) We discussed and coded the remaining uncoded responses. We also refined the initial components throughout the process until we reached a final set. This process resulted from the authors’ collaborative effort.
Step 6: Reporting components	Components, their definitions, and responses supporting them were written up ready for reporting. The coding was then ready for further analysis.

### 3.1 Components of self-control beliefs

Following the approach by Vugts et al. (2020), we first generated an initial list of words used in the responses which we subsequently categorized into themes and further coded into distinct self-control belief components. Specifically, we employed NVivo 14, a qualitative analysis and coding tool, to generate a comprehensive *word frequency list* of every word used in participants’ responses. Subsequently, the list was condensed by merging forms of words (e.g., “learnable” and “learned”) and synonyms (e.g., “learnable,” “trainable,” and “practicable”), yielding a condensed collection of *word themes*. The word themes were reviewed and revised through discussion by the authors.

Next, authors collaboratively distilled an initial and concise *list of self-control belief components*. More specifically, this iterative process involved identifying underlying conceptual similarities among the word themes and grouping them into broader categories, that is, components of self-control, that captured their core meaning. For example, word themes such as “learnable,” “progressive,” “genetic,” and “trait” were categorized under the component “learnability,” as they shared a common key thread regarding whether self-control can be learned and developed as a skill or is an inherently fixed trait. Although these word themes might slightly differ in meaning and emphasize different aspects of the component, they collectively contributed to its overall interpretation. Thus, the components were developed and explicitly reflected in the word themes.

Finally, all authors collaboratively reviewed and coded the original responses into the aforementioned self-control belief components over two rounds, as some responses contained multiple words whose full meaning might not be captured by a single word. While the aforementioned extraction process ensured that the components captured the core ideas of the responses, this review process improved accuracy in reflecting their full meanings. Each response was categorized into (only) one related component. Throughout this iterative process, the components underwent further refinement in light of the fact that the initial list of components captured the main themes and may have missed the nuances of each response.

To finalize the coding process, we converted the above categorical coding into numerical data for further analysis by assigning values to the above codes. Specifically, we assigned a score of 1 to each response (e.g., “*self-control is hard*”) that was coded under a particular component (e.g., “difficulty”), and a score of 0 for all other components (e.g., “agency”). Since each participant provided five responses, individual scores for each component ranged from 0 (i.e., no response was categorized into the component) to 5 (i.e., all responses were categorized into the component). A higher score indicated a greater frequency of responses related to a specific self-control belief component.

## 4 Results

### 4.1 Components of self-control beliefs

In the first step of our analyses, we extracted 409 single words used from all responses and counted their frequency using NVivo 14, reducing redundancy by eliminating repetitions. Subsequently, these 409 words were categorized into 49 word themes, from which an initial list of self-control belief components was created.

As a result of coding responses into components, 643 responses (85.7% of all responses) were covered and 107 (14.3%) responses were left uncoded for being either hard to categorize or idiosyncratic (e.g., “*Self-control is exercising the body*.”) or ambiguous (e.g.,” *Self-control is self-love.*”). This coding process led to a final list of 14 self-control belief components. Table 3 shows the 14 components, with brief definitions, examples of responses, number of codes, and corresponding percentages.

**Table 3:** Fourteen components of self-control beliefs.

Component	Brief definition	Examples of responses	Codes (*n*)	Percentage (%)
Difficulty	Beliefs about self-control being challenging, requiring effort, or becoming a habit.	Difficult, easy, effortless	112	14.93
Importance	Beliefs about the significance and value of self-control.	Important, necessary	99	13.20
Discipline	Beliefs about self-control involving discipline, willpower, and the ability to restrain impulses.	Discipline, willpower, restrain	97	12.93
Goodness	Beliefs about self-control being regarded as good or bad.	Good, positive, healthy, bad	83	11.07
Commitment	Beliefs about long-term commitment and goal of self-control.	commitment, perseverance	79	10.53
Success	Beliefs about success and achievement of self-control.	Success, useful	36	4.80
Agency	Beliefs related to an individual’s sense of control, rational decision-making, self-awareness, and the ability to justify concerning self-control.	Awareness, controllable	25	3.30
Learnability	Beliefs about whether self-control can be learned or developed as a skill, as well as views on inherent aspects of self-control.	Trainable, genetic	23	3.07
Morality	Beliefs about the moral judgments of self-control, including whether it is considered a virtue.	Virtuous, respectable	21	2.80
Conflict	Beliefs about the conflicts, dilemmas, choices, and ambivalence individuals experience about self-control.	Ambivalent, sacrifice, hesitation	20	2.67
Pleasure	Beliefs about self-control being regarded as pleasant or unpleasant.	Painful, unpleasant, annoying	17	2.27
Strategy	Beliefs about strategy of self-control such as avoiding temptations, prioritizing, and making realistic plans.	Avoidance, strategic	14	1.87
Mental	Beliefs about self-control being related to mind or action.	Mental, taking action	11	1.47
Motivating	Beliefs about self-control being motivating or depleting.	Motivating, invigorating	6	0.80

The percentage refers to the proportion of responses categorized under a specific component out of the total 750 responses. The responses were categorized in Step 5 of the coding process, during which all responses were reviewed and components were refined.

### 4.2 Variations in self-control beliefs

**Figure 1:**
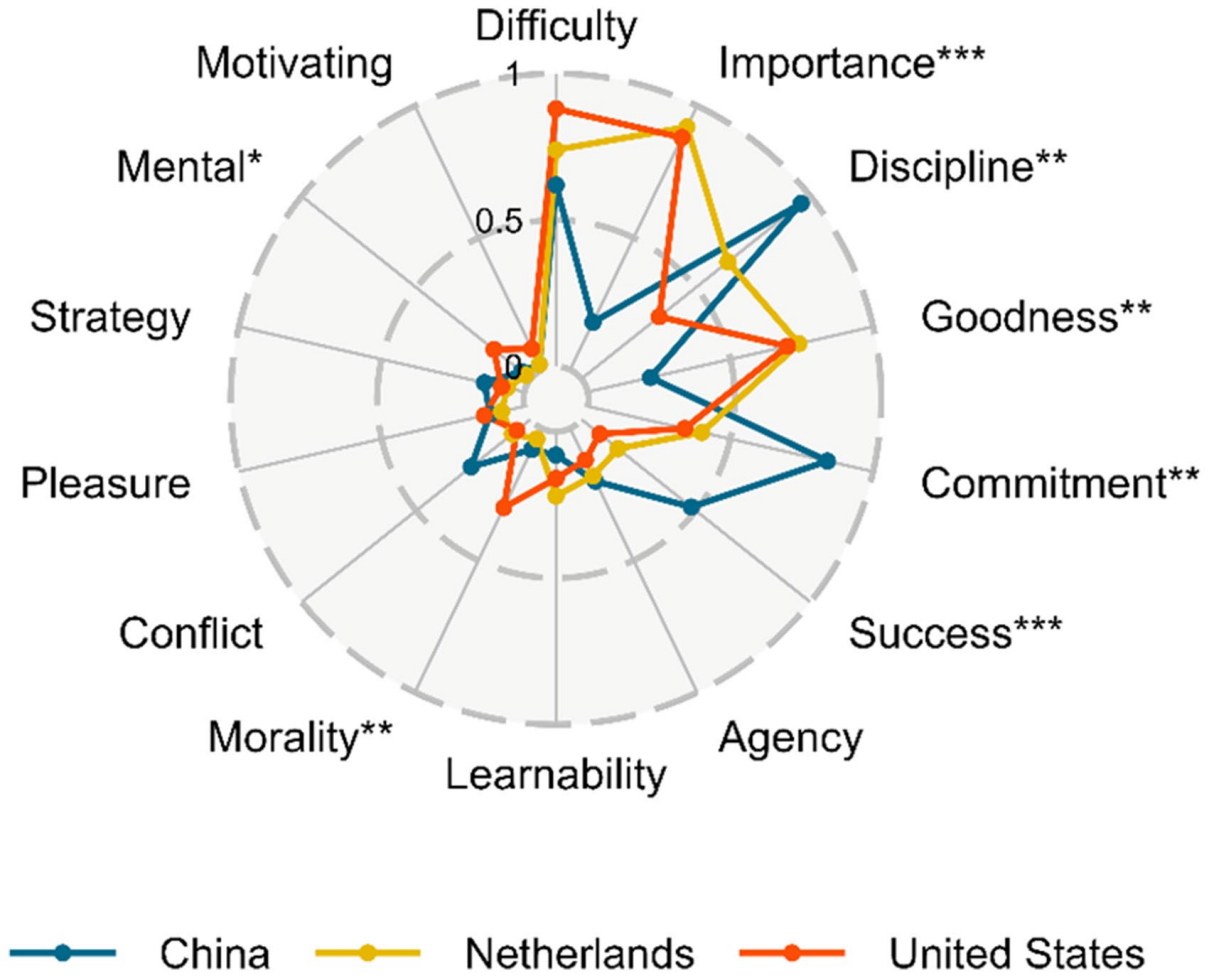
Mean scores of the fourteen self-control belief components by country. This figure shows differences in the self-control belief component scores across countries, *p < 0.05, **p < 0.01,***p < 0.001.

As shown in Figure 1, self-control beliefs varied among countries. Among the three countries, the mean value of American participants’ scores was the highest on the components “difficulty” (*M* = 0.88, *SD* = 0.94) and “importance” (*M* = 0.88, *SD* = 1.08) and the lowest on the “conflict” component (*M* = 0.06, *SD* = 0.24). Dutch participants scored highest on “importance” (*M* = 0.92, *SD* = 0.99) and had the lowest scores on the components “mental” (*M* = 0.02, *SD* = 0.14) and “motivating” (*M* = 0.02, *SD* = 0.14). Chinese participants, scored highest on “discipline” (*M* = 0.96, *SD* = 1.05) and lowest on “motivating” (*M* = 0.02, *SD* = 0.14). Significant cross-country differences were found in the following components, using Kruskal-Wallis one-way ANOVAs with country as a predictor and components scores as dependent variables: “importance” (*p* < 0.001), “discipline” (*p* = 0.002), “goodness” (*p* = 0.002), “commitment” (*p* = 0.001), “success” (*p* < 0.001), “morality” (*p* = 0.002), and “mental” (*p* = 0.036). Further *post hoc* tests showed that, overall, Chinese participants scored lower on “difficulty,” “importance,” and “goodness,” but higher on “commitment,” and “success,” than both American and Dutch participants. In contrast, Americans scored higher on “morality” and on “mental.” Appendix 1 present the full statistics.

For relationships between trait self-control and self-control beliefs, the Spearman correlation test found that only the component “success” displayed a modest yet significant correlation with trait self-control (*r* = 0.18, *p* = 0.027); the other components were not significantly related to trait self-control. Furthermore, the self-control belief components did not differ among participants with varying trait self-control levels, the high level (*N* = 54, top 34% of participants scored highest on trait self-control), the middle level (*N* = 61, middle 33% of participants), and the low level (*N* = 35, bottom 33% of participants), according to additional analysis of the Kruskal Wallis test.

Finally, three non-parametic Mann–Whitney U tests were performed to examine variances in self-control beliefs among different genders, age groups, and education levels. A significant gender difference was observed only in the component “importance,” Mann–Whitney *U* = 2131.5, *p* = 0.01. Specifically, the medium value of male participants’ scores on “important” (*Mdn* = 0.5) was significantly higher than females (*Mdn* = 0). No significant difference was found between younger (18–44 years, *N* = 137) and elder age groups (45 years and older, *N* = 13) (The Whoqol Group, 1998). Among education levels, participants’ scores differed in components including “importance” (Mann–Whitney *U* = 1420.5, *p* = 0.005), “commitment” (Mann–Whitney *U* = 2,403, *p* = 0.021), “success” (Mann–Whitney *U* = 2,354, *p* = 0.015), “morality” (Mann–Whitney *U* = 1693.5, *p* = 0.03), and “mental” (Mann–Whitney *U* = 1764.5, *p* = 0.031). Participants with the highest education level of high school tended to score higher (*Mdn* = 1) than participants graduated from college (*Mdn* = 0) in “importance.” As for “commitment,” “success,” “morality,” and “mental,” despite both groups having the same median score (Mdn = 0), the distribution of scores differed between the groups.

## 5 Discussion

Self-control beliefs are gaining attention for their potential effects on people’s self-control performance (Gennara et al., 2023; Job et al., 2010). However, a systematic investigation of these beliefs is still missing. The current study aimed to comprehensively map self-control beliefs held by people with different cultural backgrounds. We used a bottom-up approach to assess the beliefs and found that people hold multiple beliefs about self-control which could be identified into 14 main components. In doing so, our study provides a comprehensive framework for understanding the nature of self-control and its potential for enhancing and improving self-control exertion.

The present research expands upon previous research of self-control beliefs, that primarily centered upon the malleable (Mukhopadhyay and Johar, 2005) and depleting nature of self-control (Francis et al., 2023; Job et al., 2015; Savani and Job, 2017). While these belief components were also identified in our study, they were not the most prevalent. Rather, our findings showed that people placed priority on self-control’s difficulty, importance, commitment, discipline, morality, and pleasure. These foci partially overlap with a recent study by Vaughn and Burkins (2023), where researchers investigated people’s lay beliefs about self-control using linguistic methods. Their research found that people often associated self-control with its values and utility as well as with perceived emotions and feelings by analyzing words people used to describe their self-control experiences based on a dictionary commonly used in text analysis. Whereas our approach differs in that we examined self-control beliefs from a more bottom-up approach, our results point in the same direction, attesting to their robustness. We open-coded people’s thoughts about self-control without presupposition, thus revealing a comprehensive set of self-control belief components.

In contrast to previous studies on self-control beliefs that highlight the motivating and/or depleting nature of self-control, the current studies reveal that such notions are not predominant in people’s minds. One possible explanation for this difference is that people rarely spontaneously consider the resources and the depleting nature of self-control, no matter when thinking about self-control in general or when faced with specific self-control conflicts. Future studies could further examine whether these latter beliefs were non-existent or less prominent because of the self-report method we employed. It may be that these beliefs exist more implicitly, as previously observed for intelligence (Dweck and Leggett, 1988), emotion (Tamir et al., 2007), and morality (Chiu et al., 1997).

Our results confirm that individuals frequently regard self-control as requiring discipline and willpower. Willpower, commonly understood as the ability to resist unwanted behaviors or desires, is often equated with self-control. However, more recent research suggests that self-control may not solely depend on willpower, but can also result from various strategies, such as avoiding temptations or making plans (Duckworth et al., 2018; Katzir et al., 2021). Nevertheless, our findings, consistent with recent studies (e.g., Gennara et al., 2023), indicate that people tend to associate self-control more with willpower than with strategy. Strategies do not seem to be central to people’s concept of self-control (Bermúdez et al., 2023). This suggests a gap between scientific understandings of self-control and people’s lay beliefs about it.

Interestingly, while the research field has been moving away from discipline as a core theme toward a more “smart” self-control approach, emphasizing effortless exertion (Galla and Duckworth, 2015; Gillebaart and de Ridder, 2015), strategy use (Duckworth et al., 2016; Milyavskaya et al., 2021), flexibility (Bürgler et al., 2021; Werner and Ford, 2023), and metacognition (Fujita et al., 2024b; Hennecke and Bürgler, 2023), this shift does not yet seem fully reflected in people’s beliefs about self-control. Given that popular scientific books frequently talk about willpower, rather than strategy, one can wonder whether these beliefs are “innate” or (also) the result of popular scientific discourse. As such, a lag effect seems to appear in the influence of psychological research on people’s self-views (Herman, 1995).

In addition, the current study also indicates that individuals share a similar set of self-control beliefs across cultures, though they may slightly differ in the prevalence of certain components. As suggested in previous research, these differences may be an consequence of culture (Li et al., 2018; Sun et al., 2019; Yanaoka et al., 2022). Our findings show that American and Dutch participants exhibited more similar self-control beliefs, whereas Chinese participants demonstrated greater differences, particularly in emphasizing “discipline,” “commitment,” and “success.” This seems to mirror the differences in general cultural components as framed by Hofstede (2011), where China is regarded as a society with a huge attention to success and achievement. This also aligns with specific cultural traditions that discipline and restraint (*Keji* in Chinese) are especially trained as a code of conduct from one’s childhood in the Confucian society (Yun et al., 2016). Likewise, the high association between self-control and morality among American participants may reflect the influence of Protestant traditions, which historically link self-control to constraints. In this context, temperance are often regarded as virtues tied to chastity (Bermúdez et al., 2023; McCullough and Willoughby, 2009).

Lastly, our study explored whether people’s self-control beliefs are related to their actual self-control ability (i.e., trait self-control). However, findings indicate that self-control beliefs were only weakly or not at all associated with trait self-control. While previous research has suggested links between the two, no clear conclusions have been obtained (Gennara et al., 2023; Hagger et al., 2019). The non-significant relationships found in the current study might result from the bipolar responses in each component. As a consequence, it may be difficult to reveal clear directional relations between the components and trait self-control. Alternatively, it is possible is that people’s beliefs may indeed not relate to trait self-control as beliefs reflect more commonly shared ideas, thoughts, and knowledge, such as seeing it as important or effortful, regardless of a person’s actual self-control ability.

Another possibility is that self-control beliefs may be more closely related to how people manage self-control in specific situations, rather than to broad, stable tendencies captured by trait self-control measures. People with similar trait levels may hold very different beliefs about the nature of self-control, while those with similar beliefs may differ widely in their actual self-control ability.

In addition, the self-report scale we used to measure trait self-control, despite its widespread use, is known to be vulnerable to social desirability bias (Tangney et al., 2004), which may have further limited our ability to detect robust associations. Future research could benefit from a multi-method approach and using more accurate and unbiased assessments of self-control (Duckworth and Kern, 2011). For example, combining self-reports with behavioral tasks, informant ratings, or ecological momentary assessment, may obtain a more comprehensive understanding of self-control capacity.

### 5.1 Strengths and limitations

The current study successfully uncovered a comprehensive array of lay beliefs that people hold about self-control and confirmed that these beliefs were captured in people’s daily self-control conflicts. Distinct from previous research about self-control beliefs, this study featured a bottom-up approach, which yielded high ecological validity and contributed to the balance of the field.

Nevertheless, we acknowledge certain limitations of our study that should be borne in mind when interpreting results. One notable limitation is the small number of responses for some belief components, which constrains further analysis regarding beliefs and other variables. Therefore, we urge caution in interpreting these results. Additionally, unintended bias in educational background and ages across countries may have influenced the cross-country findings. Moreover, the analysis of the current study was based on translated data, which may not be as accurate as the original language due to subtle differences in connotations across languages. We also acknowledge that a certain degree of subjectivity was inevitable in creating coding schemes and the final list of components. Therefore, these should not be viewed as fixed or definitive. Instead, we present them as a foundational framework intended to guide and inform future research.

In addition, we asked participants to describe “what self-control is” in a single word. While this approach allowed us to collect responses that were on-topic, relatively structured, and less noisy, it may have constrained more complex thoughts that participants found difficult to express in just one word, especially when compared to fully open-ended responses. It may also have introduced ambiguity, as the meaning of a single word can vary across contexts. Furthermore, this method may have increased the risk of social desirability bias, as participants might have been inclined to choose words they often heard or learned, especially those aligning with commonly accepted social norms.

Finally, despite efforts to avoid influencing participants’ answers, this possibility cannot be entirely eliminated. For instance, although participants reported on beliefs about self-control as a routine or strategic, the provided self-control dilemmas may have constrained their responses, limiting their focus on more proactive or strategic approaches, such as conflict avoidance and self-distraction, which typically occur before a conflict. From this perspective, while the findings provide insight into more general self-control beliefs, they may also limit the exploration of the variations in self-control beliefs that specifically occur during or after conflicts.

### 5.2 Future directions

The current study provides a comprehensive understanding of people’s beliefs about self-control, identifying fourteen main components of these beliefs. Given the growing attention on various aspects of people’s perceptions of self-control, such as lay beliefs and self-control metacognition (Fujita et al., 2024a; Hennecke and Bürgler, 2023), we expect future research to explore diverse beliefs further. It would be intriguing to investigate how these beliefs may interact with other cognitive processes and perceptions related to self-control, including metacognition. Furthermore, examining how these lay beliefs influence individuals’ actual self-control behaviors and well-being (Bernecker et al., 2017), as well as exploring the relationships between self-control beliefs and other crucial self-control elements, such as trait self-control and state self-control, holds promise for advancing our understanding of self-control.

Moreover, it is crucial to develop an effective tool for comprehensively assessing these self-control beliefs. Existing assessments (Job et al., 2010; Mukhopadhyay and Johar, 2005) often evaluate only a limited number of beliefs. Additionally, these scales may not always meet high standards of quality (Sun et al., 2019).

Drawing from cross-cultural studies, more in-depth investigations across various cultural and social backgrounds could illuminate how lay beliefs may interact with these contexts. Furthermore, we expect more future studies of high ecological validity. This could include studies employing an experience-sampling design, diary studies, and experiments with diverse stimuli. Such approaches would provide richer insights into the complex dynamics of self-control beliefs in everyday life, for instance, about how lay beliefs may fluctuate before, among, and after self-control conflicts and how the beliefs may appear in real life contexts.

## 6 Conclusion

To comprehensively map people’s beliefs about self-control, we took a bottom-up approach to investigate, with 14 main components of self-control beliefs identified. This includes difficulty, importance, discipline, goodness, commitment, success, agency, learnability, morality, conflict, pleasure, strategy, mental, and motivating. The components were observed in discussion of self-control across three cultures. Findings contribute to an in-depth understanding of how self-control is viewed and believed. Exploration of self-control theories beyond the traditional view of limited resources could offer valuable insights and inspire future research in this field.

## Data Availability

The datasets presented in this study can be found in online repositories. The names of the repository/repositories and accession number(s) can be found at: https://osf.io/27jte/.

## Footnotes

^1^ https://osf.io/27jte/

^2^The present study is partly inspired by a pilot study examining self-control beliefs with a slightly different method. Materials and results are available at OSF.

^3^Twenty responses were selected randomly and translated by both ChatGPT 3.5 and experts fluent in both languages. Results showed over 95% consistency between ChatGPT 3.5 and experts. Besides, translated responses that could not be understood well were rechecked by experts in the later analysis.

## Appendix: Pairwise comparisons of cross-country differences in self-control belief components

Kruskal-Wallis one-way ANOVAs revealed significant cross-country differences in several components, including “importance” (*p* < 0.001), “discipline” (*p* = 0.002), “goodness” (*p* = 0.002), “commitment” (*p* = 0.001), “success” (*p* < 0.001), “morality” (*p* = 0.002), and “mental” (*p* = 0.036). We then conducted post hoc comparisons to examine pairwise differences between countries on these components. As shown in Table A1, Chinese participants reported significantly lower scores on “difficulty,” “importance” and “goodness,” but significantly higher scores on “commitment” and “success,” compared to both American and Dutch participants. They also reported higher scores on “discipline” than American participants. In contrast, American participants reported significantly higher scores on “morality” compared to both Chinese and Dutch participants, and higher scores on “mental” compared to Dutch participants.

**Table A1:** Pairwise comparisons of cross-country differences in self-control belief components.

Pairs	Importance	Discipline	Goodness	Commitment	Success	Morality	Mental
CN-US	4.08^***^	−3.48^**^	2.89^*^	−2.96^**^	−4.37^***^	2.74^*^	2.01
CN-NL	4.65^***^	−1.24	3.24^**^	−3.36^**^	−3.42^**^	−0.59	−0.40
US-NL	−0.58	−2.24	−0.35	−0.40	−0.95	3.33^**^	2.41^*^

(a) ^*^Bonferroni adjusted *p* < 0.05, ^**^ < 0.01, ^***^ < 0.001; (b) CN = China, US = United States, NL = the Netherlands.
